# Local Diagnostic Reference Levels for Common Nuclear Medicine Procedures for Pediatric in Dubai Health

**DOI:** 10.3390/pediatric18010021

**Published:** 2026-02-03

**Authors:** Entesar Z. Dalah, Najlaa K. Al Mazrouei, Zahra A. Al Ali

**Affiliations:** 1 Central Diagnostic Imaging Department, Dubai Health, Dubai P.O. Box 2727, United Arab Emirates; 2 College of Medicine, Mohammed Bin Rashid University, Dubai Health, Dubai P.O. Box 2727, United Arab Emirates; 3 Medical Physics Department, Dubai Hospital, Dubai Health, Dubai P.O. Box 2727, United Arab Emirates; 4 Nuclear Medicine Center, Dubai Hospital, Dubai Health, Dubai P.O. Box 2727, United Arab Emirates

**Keywords:** pediatric nuclear medicine, diagnostic reference levels (DRLs), scintigraphy nuclear medicine, hybrid PET/CT, Dubai Health

## Abstract

This study aims to establish diagnostic reference levels (DRLs) for common pediatric nuclear medicine (NM) procedures performed within the Dubai Health sector. The established DRLs will serve as a benchmark for pediatric NM practice, supporting standardized healthcare delivery and guiding ongoing quality improvement and internal audit activities. Patient dose survey data were collected from the solo NM center within the Dubai Health sector. The study included common scintigraphy procedures using gamma cameras and the hybrid positron emission tomography with computed tomography (PET/CT) procedures. Scintigraphy procedures include the dynamic and static renal scans, and ocular eye scans. The hybrid PET/CT procedures entail tumor/infection and neuroendocrine scans. Patient demographics, administered activities, CT doses, and study description were recorded. Both weight bands of <5, 5–<15, 15–<30, 30–<50, and 50–<80 kg, and age bands of <1, 1–<5, 5–<10, and 10–<15 years were considered. Statistical analysis was performed to determine the 25th percentile, median and 75th percentile of the dose distribution. The median value was used to establish the DRLs for the Dubai Health sector. The analyses revealed significant variation in the administered activities across the different pediatric NM procedures. The proposed DRLs for various pediatric NM procedures for the weight band 15–<30 kg are as follows: renal dynamic 98.4 MBq, renal static 96.2 MBq, ocular eyes 18.5 MBq, tumor/infection 155 MBq, and neuroendocrine 80 MBq. This work provides the first pediatric NM DRLs for the Dubai Health sector, offering a key reference for developing the local DRLs for the Emirate of Dubai. The findings indicate that achieving meaningful dose optimization will require systematic revision of existing imaging protocols, with targeted parameter adjustments informed by continuous dose monitoring and benchmarking to enhance patient safety and overall diagnostic quality.

## 1. Introduction

Pediatric patients are particularly vulnerable to ionizing radiation due to their developing tissues and longer life expectancy [1,2], which increases their potential for radiation induced risks [3]. Nuclear medicine (NM) procedures involve administering radiopharmaceuticals to diagnose and treat various conditions, offering diagnostic information that is often unobtainable with other imaging modalities [4]. Computed tomography (CT) may be required in specific NM procedures to support attenuation correction and localization, as well as to enable spatial tumor characterization in conjunction with positron emission tomography (PET) imaging. Consequently, the radiation exposure to the pediatric patient is increased [5]. In association with the above, optimizing radiation doses in pediatric NM procedures is of paramount importance.

Diagnostic reference levels (DRLs) are established benchmarks that support the management and optimization of patient radiation doses, ensuring that radiation exposure remains as low as reasonably achievable (ALARA) while maintaining diagnostic efficacy [6,7]. In NM, scanning protocols must be carefully designed to balance image quality with dose optimization, incorporating strategies such as weight based administered activity and tailoring CT parameters, particularly for pediatric patients.

DRLs are not dose limits but serve as investigational tools to identify unusually high or low radiation doses for a given procedure [8]. International Commission on Radiological Protection (ICRP) report 135 [8] recommends using routinely available clinical quantities (administered activity and CT dose indices). Healthcare facilities are encouraged to compare their practices with national or regional DRLs to identify opportunities for dose optimization, ensure consistent high quality patient healthcare service, and take corrective actions when deviations are identified. This process supports the broader goal of justification and optimization in medical imaging, as outlined in ICRP report 135 [8]. ICRP 135 does not recommend using effective dose for DRLs and instead requires measurable quantities at the time of the procedure. Compared to adults, several challenges are faced while performing NM procedures for the pediatric population, particularly the need to amend the scan acquisition parameters and the administered activity is required. International guidelines, such as those provided by the ICRP [8] and the European guidelines on DRLs for pediatric imaging, report 185 [9], offer valuable frameworks for establishing and implementing DRLs in pediatric radiology.

These guidelines emphasize the importance of using appropriate dose quantities that account for patient weight or age to ensure effective dose management. Number of resources are available to assist in obtaining high quality NM images at low administered radiopharmaceuticals. Examples include the latest version of the European Association of Nuclear Medicine (EANM) dose card [10] and the North American Consensus guidelines for administered radiopharmaceutical activities in pediatric [11], in addition to the dose calculator provided by the Society of Nuclear Medicine and Molecular Imaging (SNMMI) based on [10,12,13]. All above mentioned guidelines recommend adjusting the administered activity based on the patient’s weight, ensuring that the maximum administered activity does not exceed the product of the patient’s weight and the recommended weight based administered activity [11]. It is well known that pediatric patients vary significantly in size and physiology, hence age based administered activity is not sufficient for dose optimization. Considering different weight bands provides a more accurate method for grouping pediatric patients when establishing DRLs, especially for procedures involving ionizing radiation like NM and CT [8,9].

Despite the acknowledged benefits and existence of DRLs for pediatric, there is a lack of reported DRLs that are standardized based on weight bands, particularly those specifically tailored for pediatric NM procedures [8,14]. This gap highlights the need for radiation dose surveys to focus on establishing weight band pediatric specific DRLs, which can guide practitioners in optimizing radiation doses for young patients. This local patient dose review aims to address this need by analyzing data from our solo governmental NM center in Dubai Health sector, and to establish preliminary weight bands as well as age bands pediatric specific DRLs for the common pediatric NM procedures. Weight based DRLs are physiologically more accurate. This dose survey review fills a global evidence gap by providing weight stratified DRLs from a region with no prior data.

Further, the correlation between the administered activity based on weight bands versus age bands will be demonstrated.

## 2. Materials and Methods

Patient administered activity, radiopharmaceutical, and CT dose data utilized in this healthcare sector dose report, received approval from the Dubai Scientific Research Ethics Committee (DSREC) at the Dubai Health Authority on 14 August 2024. Data collection encompassed patient CT dose surveys and radiopharmaceutical administered activity measurements conducted between 1 January and 31 December 2024. The datasets needed were obtained retrospectively through systematic retrieval using the electronic radiation dose monitoring and tracking system, DOSE TQM (Qaelum N.V. 19v) [15]. This facilitated identifying and analyzing the most frequently (also known as common) performed scintigraphy and hybrid NM procedures among pediatric patients at our dedicated NM center. Our center is facilitated with two gamma cameras, both GE Discovery 630 NM (GE Healthcare, Waukesha, WI, USA), a hybrid single photon emission tomography with a CT (SPECT/CT) scanner (GE Discovery 670, 16 slices), and a hybrid positron emission tomography with CT (PET/CT) scanner (GE Discovery MI DR, 64 slices). Patient demographic and procedural information, including age, weight, study description, anatomical region examined, radiopharmaceuticals administered, NM modality, administered activity, CT dose and CT acquisition parameters, were systematically collected through the DOSE TQM platform.

In the context of gamma camera and SPECT imaging, 99m-technetium (^99m^Tc) remains the predominant isotope, combined with various pharmaceuticals to produce specific radiopharmaceuticals. Pediatric scintigraphy procedures that were performed at our NM center and retrieved in this survey are: ^99m^Tc-dimercaptosuccinic acid (^99m^Tc-DMSA) for static renal scans, ^99m^Tc-mercaptoacetyltriglycine (^99m^Tc-MAG3) for dynamic renal function assessments (renograms), ^99m^Tc-pertechnetate for dacryoscintigraphy (ocular assessment), ^99m^Tc-hydroxydiphosphonate (^99m^Tc-HDP) for bone scans, ^99m^Tc-microaggregated albumin (^99m^Tc-MAA) for lung scans, and ^99m^Tc-pretechnetate tagged red cells for Meckel’s diverticulum scan. For the hybrid NM procedures, specifically PET/CT imaging, the prevalent studies are, whole-body (WB) PET using ^18^F-fluorodeoxyglucose (^18^F-FDG) for oncology and infection evaluation and Gallium-68 DOTATOC (^68^Ga-DOTATOC) for neuroendocrine tumor diagnosis. The hybrid CT component of PET/CT procedures primarily involves low dose CT for attenuation correction and anatomical localization during WB PET scans. Radiation dose metrics, including volumetric CT dose index (CTDI_vol_, in mGy) and dose-length product (DLP, in mGy·cm), were recorded for these procedures.

As this survey involved a single center providing nuclear medicine services within the Dubai Health sector, and in accordance with ICRP 135 [8] guidance for similar contexts, DRLs for each nuclear medicine procedure were derived using the median values of the collected dose distributions for commonly performed scintigraphy and hybrid NM examinations. While ICRP 135 [8] recommends a sample size of approximately 30 examinations for establishing DRLs in adult CT studies, no specific minimum is prescribed for pediatric imaging. Given the substantially lower examination frequency and broader age and weight-based stratification in pediatric practice, the achievable sample size is often smaller. Accordingly, the small pediatric sample size in this study reflects real clinical throughput and remains consistent with ICRP 135 [8] guidelines for dose audits in low volume pediatric populations. Patient dose indicators are also presented at the 25th and 75th percentile to facilitate benchmarking and quality assurance. To support internal auditing and optimization, data were stratified into five weight band groups following the ICRP 135 and European guidelines [8,9], including: <5, 5–<15, 15–<30, 30–<50, 50–<80 kg. To facilitate aligning our own pediatric specific DRLs with international and national existing DRLs, age bands were also considered. Four age band groups were identified: <1, 1–<5, 5–<10, 10–<15 years [8,9].

### Statistical Analysis

Statistical analysis was conducted using GraphPad Prism 8, V8.03, GraphPad Software, San Diego, CA, USA. Quantitative variables are expressed as the median (50th), 25th and 75th percentiles. The Spearman coefficient test was used for correlation analysis. The non-parametric Spearman coefficient test handles outliers better than the parametric Pearson coefficient test. Kruskal–Wallis and Mann–Whitney tests were used for significance. *p* = 0.05 was used for significance.

## 3. Results

### 3.1. Survey Sample

Dose survey for a total of 258 (178 scintigraphy including the two gammas and SPECT, and 37 PET/CT) pediatric NM procedures performed over a 12 month period was collected and retrospectively analyzed. The most common scintigraphy NM procedure registered for pediatrics over the stated period was renogram, a dynamic renal function assessment using ^99m^Tc-mercaptoacetyltriglycine (^99m^Tc-MAG3) (sample size, 102), followed by the renal static scan using ^99m^Tc-dimercaptosuccinic acid (^99m^Tc-DMSA) (sample size, 53). The most common PET/CT procedures performed were WB using ^18^F-FDG (sample size, 21) and ^68^Ga-DOTATOC (sample size, 16). Scintigraphy studies such as bone, lung, and Meckel’s scans were excluded due to insufficient sample size (total sample size, 23). Similarly, the CT data of the hybrid SPECT were also excluded from this dose review due to the limited sample size (total sample size, 6). In this study, procedures with a sample size of fewer than 10 were excluded to ensure meaningful statistical analysis.

### 3.2. CT Acquisition Parameters

For the CT of the hybrid PET/CT, the machine default acquisition parameters for the WB scan are demonstrated in Table 1. Fundamentally, the high preset pitch results in a lower CT dose scan. Smart tube current (mA), also referred to as tube current modulation, was enabled for all CT scans in the present study, providing individualized optimization. However, a fixed tube potential kilovoltage (kVp) was applied for patients undergoing PET/CT examinations. An adaptive statistical iterative reconstruction (ASiR) algorithm was used for the CT images.

### 3.3. Weight vs. Age Impact

#### 3.3.1. Scintigraphy Procedures

**Figure 1:**
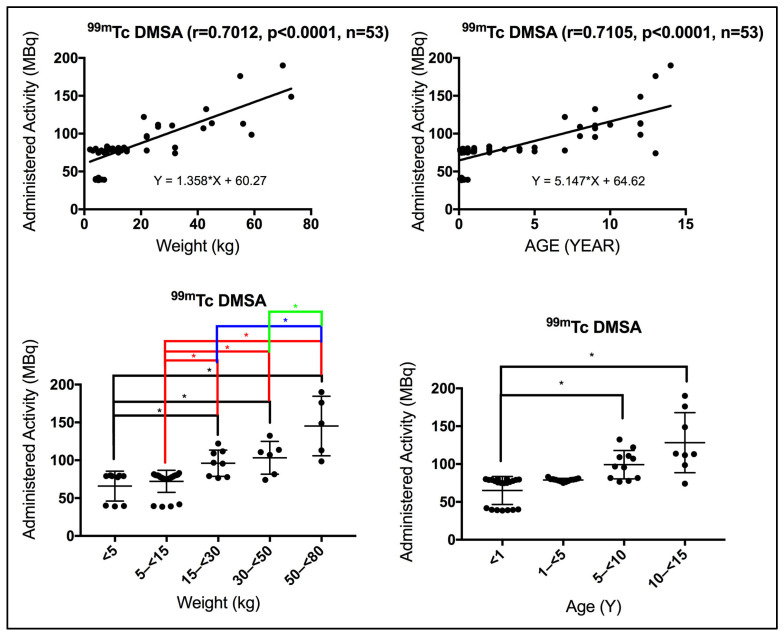
(**Top** panel) Strong correlation was observed between patient weight/age and the administered activity in renal static scintigraphy. r denotes the Spearman correlation coefficient, *p* < 0.05 denotes significance, and n denotes the sample size. (**Bottom**) Administered activity based on weight and age bands varies significantly. Using the Kruskal–Wallis test, weight and age bands show significant differences in administered activity, annotated by * (*p* < 0.05).

Using the Spearman correlation coefficient test, a positive strong significant correlation was seen between patient weight and the administered activity for patients subjected to renal static scan using ^99m^Tc-DMSA (r = 0.7012, *p* < 0.0001). Similarly, a positive strong significant correlation was also seen between patient age and the administered activity for patients subjected to renal static scan using ^99m^Tc-DMSA (r = 0.7105, *p* < 0.0001). However, a slightly stronger correlation was seen with age bands in contrast to weight bands (Figure 1), implying that the administered activity reviewed in this survey was not strictly weight band dependent. Using the Kruskal–Wallis test, significant differences were seen in the administered activity between the different weight and age group bands, as annotated by *, Figure 1.

**Figure 2:**
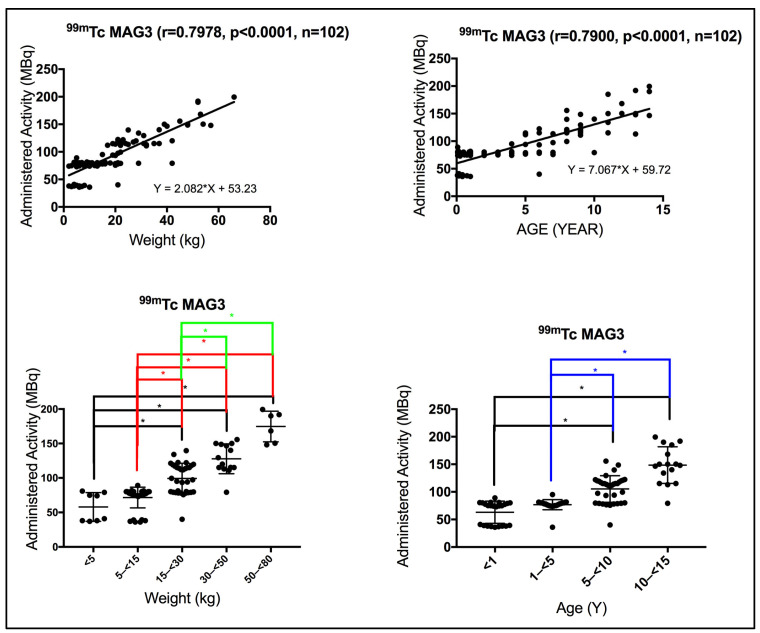
(**Top** panel) Strong correlation was observed between patient weight/age and the administered activity in dynamic renal scintigraphy. r denotes the Spearman correlation coefficient, *p* < 0.05 denotes significance, and n denotes the sample size. (**Bottom** panel) Administered activity based on weight and age bands varies significantly. Using the Kruskal–Wallis test, weight and age bands show significant differences in administered activity, annotated by * (*p* < 0.05).

Using the Spearman correlation coefficient test, a positive, strong, and significant correlation was seen between patient weight and the administered activity for patients subjected to dynamic renal scan (renogram) using ^99m^Tc-MAG3 (r = 0.7978, *p* < 0.0001). Likewise, a positive, strong, and significant correlation was also seen between patient age and the administered activity for patients subjected to dynamic renal scan (renogram) using ^99m^Tc-MAG3 (r = 0.7900, *p* < 0.0001). In contrast to the renal static scan, the administered activity appeared to be predominantly weight dependent, as indicated by the slightly higher correlation compared to age-based activity, Figure 2. Using the Kruskal–Wallis test, significant differences were seen in the administered activity between the different weight and age group bands, as annotated by *, Figure 2.

#### 3.3.2. PET of the Hybrid PET/CT Procedures

**Figure 3:**
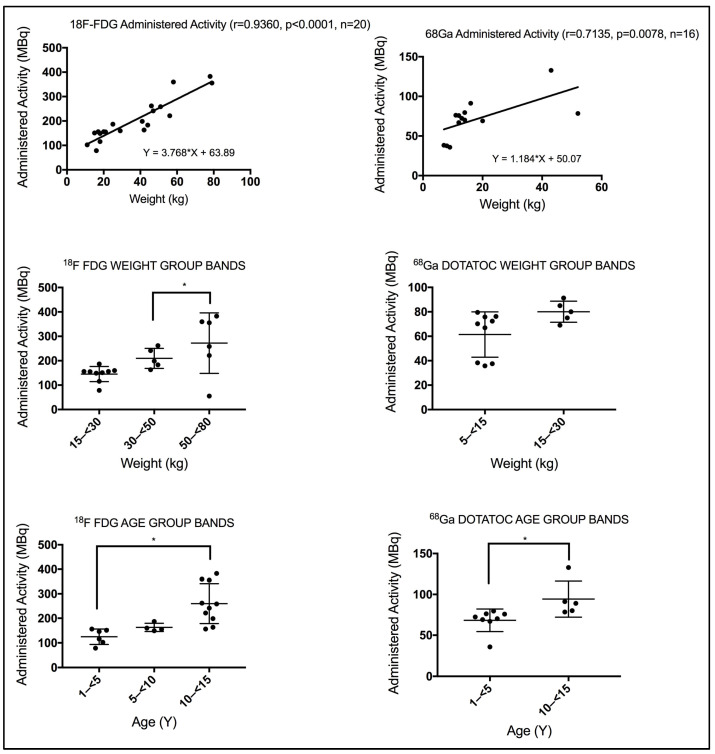
(**Top** panel) A strong correlation was observed between patient weight and the administered ^18^F-FDG activity compared with ^68^Ga PET/CT procedures. r denotes the Spearman correlation coefficient, *p* < 0.05 denotes significance, and n denotes the sample size. (**Bottom** panel) Administered activity based on weight and age bands varies significantly. Using the Mann–Whitney test, weight and age bands show significant differences in administered activity, annotated by * (*p* < 0.05).

Using the Spearman correlation coefficient test, a strong significant correlation was seen between patient weight and the administered activity for patients subjected to ^18^F-FDG WB (r = 0.9360, *p* < 0.0001). The ^68^Ga-DOTATOC procedure also disclosed a strong significant correlation (r = 0.7135, *p* = 0.0078). Using the Kruskal–Wallis test, significant differences were seen in the administered activity between the different weight and age group bands for patients subjected to ^18^F-FDG WB, as annotated by *, Figure 3. In contrast, insignificant differences were seen in the administered activity between the two weight band groups for patients subjected to ^68^Ga-DOTATOC procedures using the Mann–Whitney test, Figure 3. Whereas, differences exist in the administered activity between the two age band groups for patients subjected to ^68^Ga-DOTATOC procedures using the Mann–Whitney test, Figure 3.

#### 3.3.3. CTDI_vol_ of the Hybrid PET/CT Procedures

**Figure 4:**
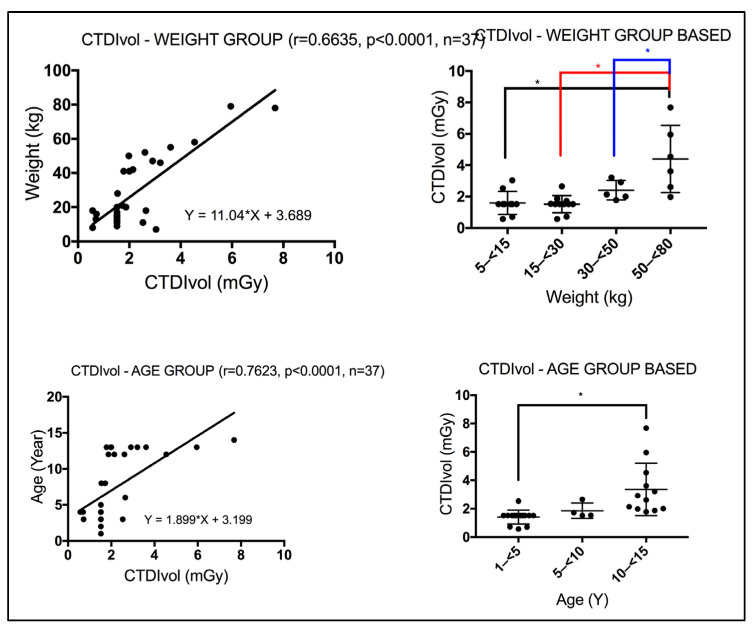
(**Left**-side panel) A stronger correlation was observed between age and the CTDI_vol_ in patients undergoing WB CT scan compared with weight. r denotes the Spearman correlation coefficient, *p* < 0.05 denotes significance, and n denotes the sample size. (**Right**-side panel) CTDI_vol_ based on weight and age bands varies significantly. Using the Kruskal–Wallis test, weight and age bands show significant differences in CTDI_vol_, annotated by * (*p* < 0.05).

For the hybrid CT of the PET/CT, a moderate significant correlation was seen between weight and the CTDI_vol_ for patients subjected to WB CT scans (r = 0.6635, *p* < 0.0001) using a Spearman correlation coefficient test. In contrast, a strong significant correlation was seen between age and the CTDI_vol_ for patients subjected to WB CT scans (r = 0.7623, *p* < 0.0001), Figure 4. Using the Kruskal–Wallis test, significant differences were seen in the CTDI_vol_ between the different weight and age group bands for patients subjected to WB CT scans, as annotated by *, Figure 4. WB CT scans include the torso extending to the pelvis region. The CTDI_vol_ in this anatomical region is known to be affected by weight. Using linear regression analysis, AlShurbaji et al. [16] studied the impact of CT scan acquisition and patient related factors on CT dose, reporting a significant correlation between patient weight and CTDI_vol_ across their investigated CT exams (chest, cardiac, abdomen, and pelvis).

### 3.4. Diagnostic Reference Levels, DRLs

#### 3.4.1. DRLs for the Administered Activity for Scintigraphy and PET Procedures

Summarized ranges (minimum and maximum) of administered activity classified per weight bands for the common scintigraphy and hybrid PET/CT procedures carried out in our NM center are presented in Table 2. According to the updated North American Consensus Guidelines for pediatric administered radiopharmaceutical activity, as published by Treves et al. [11], the administered activity per weight for an ^18^F-FDG body scan ranges from 3.7 to 5.2 MBq/kg, with a minimum recommended activity of 26 MBq. In the present review, we report a minimum of 55 MBq. For ^68^Ga-DOTATOC, the recommended administered activity is 2.7 MBq/kg, with suggested minimum and maximum values of 14 MBq and 185 MBq, respectively. In our data, we report a minimum of 35.8 MBq and a maximum of 91.3 MBq. For the renal static scan using ^99m^Tc-DMSA, the recommended administered activity is 1.85 MBq/kg, with minimum and maximum values of 18.5 MBq and 100 MBq, respectively. For pediatric patients weighing more than 70 kg, the maximum administered activity should not exceed the product of 1.85 MBq/kg multiplied by body weight. For example, in an 80 kg patient, the administered activity should not exceed 148 MBq. In this review, we report a minimum of 38.6 MBq and a maximum of 190 MBq. For the dynamic renal (renogram) scan using ^99m^Tc-MAG3, the recommended administered activity is 3.7 MBq/kg, with minimum and maximum administered activity values of 37 MBq and 148 MBq. As with the renal static scan, for patients weighing more than 70 kg, the administered activity should not exceed the product of 3.7 MBq/kg multiplied by body weight. We report a minimum of 36 MBq and a maximum of 199.4 MBq. At this point, it is worth noting that the higher administered activities observed in this review are primarily associated with the 50–<80 kg weight band, as shown in Table 2. This range overlaps with typical adult body weights, for which the recommended administered activity for renal static and dynamic scans is 185 MBq. Consequently, children whose weight is comparable to or exceeds that of an average adult (approximately 70 kg) may be assigned the adult administered activity. However, good practice dictates adherence to the most recent publications relevant to pediatric administered activity [10,11].

Median values were taken as representatives of the DRLs for the common nuclear medicine procedures at our nuclear medicine facility, as recommended by [8]. The patient activity survey for this health sector expressed as the 25th, 50th, and 75th percentiles, was calculated for each radiopharmaceutical used in scintigraphy and hybrid PET/CT procedures. Table 3 presents the DRLs stratified by weight bands. Our weight based DRLs align with ICRP 135 [8] and EANM [6] recommendation. Table 4 displays the DRLs by age bands, benchmarked against national values when available.

#### 3.4.2. DRLs for CTDI_vol_ and DLP in the Hybrid PET/CT Scans

Median values were taken as representatives of the CT DRLs in the present dose review and were determined for the WB CT scan of the hybrid PET/CT. Table 5 demonstrates the DRLs based on weight bands, and Table 6 shows the DRLs based on age bands, benchmarked against national values when available.

## 4. Discussion

To ensure the delivery of high quality pediatric radiological and nuclear medicine care, strict adherence to the core principles of radiation protection including justification, optimization, and dose limitation is essential [8,22]. Among these, the principle of optimization emphasizes minimizing radiation exposure while maintaining sufficient image quality for accurate diagnosis. This consideration is particularly critical in pediatric populations due to their increased radio-sensitivity and longer post exposure lifespan, which elevate the potential risks associated with ionizing radiation [9]. DRLs serve as vital benchmarks in dose optimization efforts, providing guidance for clinical practice by identifying potential areas for dose reduction without imposing rigid limits. DRLs facilitate quality improvement by highlighting deviations from expected practice and prompting review of protocols, equipment performance, and technical procedures such as patient positioning (centering), an element particularly relevant in pediatric CT imaging. Adherence to zero vertical and horizontal positioning offset has a significant impact on both radiation dose and image quality [23,24,25]

This review provides the first comprehensive DRL framework in our standalone NM center, including dose reference data for pediatric scintigraphy (planar or SPECT), hybrid PET/CT, and the CT component of hybrid PET/CT procedures. DRLs were established for three common pediatric scintigraphy protocols, two hybrid PET/CT procedures and the associated CT scans, integrating both administered radiopharmaceutical activities and CT dose indices (CTDI_vol_ and DLP). The dataset encompasses the entire pediatric cohort, stratified by age and weight band categories for each NM procedure. The primary aim of this dose guide is to promote safe clinical practices by facilitating dose optimization and supporting internal quality assurance initiatives [8]. The provided tables detail dose ranges categorized by age and weight band groups and include comparative analysis with international data, thereby providing a benchmark for safe and effective pediatric nuclear medicine imaging.

When comparing our proposed pediatric DRLs with those reported internationally, several patterns emerge that place our findings in a broader global context. For ^18^F-FDG PET/CT, our weight band DRLs fall within the upper range of values reported in Australia, Kuwait, and Pakistan, reflecting differences in patient habitus, protocol design, and scanner technology across regions. For ^99m^Tc-MAG3 and ^99m^Tc-DMSA, our DRLs are generally comparable to those published in neighboring Gulf countries, although variations persist due to differences in weight band definitions and institutional practices. Importantly, most published pediatric DRLs remain age-based, whereas our study provides weight stratified DRLs that align more closely with ICRP 135 [8] and EANM [6] recommendations. This distinction enhances the physiological relevance of our benchmarks and contributes unique data to the global DRL evidence base, particularly from a region where pediatric NM DRLs are limited. Collectively, these comparisons highlight both areas of alignment with international practice and opportunities for further protocol optimization within our local setting.

Based on our findings, we recommend the following steps to optimize the administered injected dose: establish clear weight group bands, preferably following those recommended in [8,9], use patient weight as the primary determinant for radiopharmaceutical activity, and ensure consistency in practice.

Evaluation of the CT component of the hybrid PET/CT imaging demonstrates that dose indices, including CTDI_vol_ and DLP, are consistently higher in our center compared to international references. The median DLP also surpasses international figures across all weight and age band groups examined. These findings highlight substantial scope for dose reduction and underscore the importance of establishing local DRLs and implementing dose optimization strategies aligned with international standards to enhance pediatric radiation safety. While current CT protocols appear conservative enough to preserve diagnostic image quality, the observed dose levels particularly when compared to Australian benchmarks warrant further inspection. Several technical and clinical factors may contribute to these elevated doses, including default protocol settings with higher tube current (mA), patient positioning, and the use of fixed tube potential voltage (kVp) rather than adaptive potential modulation technology. Additionally, clinical preferences for higher resolution imaging in complex cases may drive protocol conservatism.

The CT acquisition parameters for ^18^F-FDG and ^68^Ga-DOTATOC are typically applied to large adult patients. This may represent a potential key for CTDI_vol_ dose reduction. CTDI_vol_ dose optimization can be achieved through targeted protocol adjustments and enhanced staff technical proficiency. Examples include reducing the maximum tube mA range, enabling modulated kVp or defining a preset kVp range, particularly for smaller and thin patients, and ensuring zero vertical and horizontal positioning offset. Accurate patient positioning in pediatric CT is fundamental to dose optimization, as it ensures the targeted anatomy is properly aligned within the scan field, thereby reducing the necessity for repeat scans and the subsequent radiation exposure that comes with it [24]. In addition, the use of machine learning (ML)-based reconstruction algorithms may further improve dose efficiency due to their well-established noise handling capabilities. These measures can reduce radiation exposure without compromising diagnostic efficacy. Such opportunities should be examined in future internal and multicenter audits to support alignment with international standards while preserving clinical confidence.

In alignment with ICRP publication 135 [8], it is important to clarify that DRLs are not based on effective dose (mSv). Effective dose is a risk related quantity intended for population level comparisons and long-term epidemiological assessments and therefore is not suitable for optimization of individual clinical procedures. ICRP 135 explicitly recommends that DRLs be derived from quantities that are directly measurable at the time of the examination such as administered activity in nuclear medicine and CTDI_vol_ or DLP for hybrid CT components because these metrics reflect actual practice and can be reliably compared across institutions. For this reason, our DRLs are appropriately based on administered activity and CT dose indices, consistent with international guidance. Incorporating effective dose into DRL establishment would not align with ICRP recommendations and would introduce unnecessary uncertainty without improving optimization outcomes.

Comprehensive staff training are imperative strategies to enhance dose management, improve patient safety, and support the overall goal of radiation protection in pediatric imaging. Limited studies disclose CT scan acquisition parameters used for adult hybrid scans, whether SPECT/CT or PET/CT, such as the examples in [5,17]. To the authors’ knowledge, no published record exists describing CT scan acquisition parameters for pediatric hybrid NM scans. This is the first weight band pediatric DRLs dataset in the country; it contributes to the global movements towards weight based DRLs. This limitation was further compounded by technical challenges encountered during data collection, particularly the difficulty in including the CT component of hybrid SPECT/CT scans due to issues faced while exporting the studies to DOSE TQM. This resulted in a limited number of procedures registered in DOSE TQM, our data source. The limited number of cases within each weight and age band, an inherent challenge in pediatric cohorts further contributes to the study’s constraints. To address these limitations and strengthen the robustness of future analyses, we strongly recommend adopting a multicenter study design. Such collaboration would enable larger, more diverse datasets, facilitate inclusion of underrepresented procedures, and support the development of more universally applicable standards.

## 5. Conclusions

The current protocols may not yet be fully optimized for dose minimization, potentially reflecting conservative practices or protocol variations aimed at maintaining image quality. A significant limitation of this study is the relatively small sample size, which restricted the robustness and generalizability of the findings. Consequently, these factors underscore the need for multicenter studies to validate these results and facilitate the development of nationally applicable pediatric DRLs that promote consistent dose optimization and quality assurance across the region.

## Figures and Tables

**Table 1:** Whole body CT acquisition parameters for the CT of the hybrid PET/CT.

Study	kVp	Smart mA	Rotation Time (s)	Noise Index	Pitch	Speed (mm/Rotation)	Slice Thickness (mm)	Detector Rows
^18^F-FDG	120	30–300	0.8	16	1.375	55	5	40
^68^Ga-DOTATOC	120	30–300	0.8	16	1.375	55	5	40

Tube current (mA); Tube potential kilovoltage (kVp).

**Table 2:** Administered activity range classified per weight band for pediatric common scintigraphy and hybrid PET/CT procedures in Dubai Health.

Scan	Clinical Indication	Radiopharmaceutical	Administered Activity (MBq) MIN–MAX				
			<5 kg	5–<15 kg	15–<30 kg	30–<50 kg	50–<80 kg
Tumor/Infection	Oncology (staging, treatment response)/Infection (fever of unknown origin, vasculitis)	^18^F-FDG	-	-	78.45–186.8	162.9–261.7	55.0–382.6
Neuroendocrine	Brain tumors, neuroendocrine malignancies, congenital hyperinsulinism	^68^Ga-DOTATOC	-	35.8–79.6	69.01–91.3	-	-
Renal dynamic	Urinary tract obstruction, Urinary leaks, Renovascular hypertension	^99m^Tc-MAG3	37.0–80.9	36.0–89.1	40.1–139.6	79.3–155.8	148.0–199.4
Renal static	Acute pyelonephritis, Assess renal function	^99m^Tc-DMSA	39.0–80.0	38.6–83.1	76.6–122.0	74.0–132.4	98.6–190.0
Ocular (Eyes)	Epiphora, Assess lacrimal duct obstructions	^99m^Tc-Pertechnetate	18.5				

**Table 3:** Administered activity DRLs based on weight bands for pediatric common scintigraphy and hybrid PET/CT procedures in Dubai Health compared with other countries.

Radiopharmaceutical (Scan)	Weight Band (kg)	Administered (MBq)			Other Countries
		25th Percentile	50th Percentile (DRL)	75th Percentile	
^18^F-FDG (Tumor/Infection)	15–<30	132.2	155.0	158.0	107.6 Australia [17]
	30–<50	173.1	198.7	251.6	150.3 Australia [17]
	50–<80	179.9	306.9	365.4	189.6 Australia [17]
^68^Ga-DOTATOC (Neuroendocrine)	5–<15	38.0	70.2	76.0	
	15–<30	72.0	80.0	88.1	
^99m^Tc-MAG3 (Renal dynamic)	<5	38.0	57.5	78.0	
	5–<15	74.3	76.6	80.0	
	15–<30	79.2	98.4	117.2	
	30–<50	114.5	124.7	149.0	
	50–<80	149.8	179.2	193.8	
^99m^Tc-DMSA (Renal static)	<5	39.8	79.0	79.3	
	5–<15	75.0	77.6	80.1	
	15–<30	78.1	96.2	111.0	
	30–<50	79.7	108.9	118.5	
	50–<80	105.8	148.8	183	

**Table 4:** Administered activity DRLs based on age bands for pediatric common scintigraphy and hybrid PET/CT procedures in Dubai Health compared with other countries.

Radiopharmaceutical (Scan)	Age Band (Year)	Administered (MBq)			Other Countries
		25th Percentile	50th Percentile (DRL)	75th Percentile	
^18^F-FDG (Tumor/Infection)	1–<5	96.2	130.8	152.3	70 Kuwait [18], 77.5 Australia [17], 82 Pakistan [19]
	5–<10	150.6	157.5	180.1	120 Kuwait [18], 116.2 Australia [17], 97 Pakistan [19], 100 [20]
	10–<15	189.8	249.9	356.5	189 Kuwait [18], 179.9 Australia [17], 102 Pakistan [19], 133 [20]
^68^Ga-DOTATOC (Neuroendocrine)	1–<5	67.5	71.3	76.1	
	10–<15	79.2	89.0	112.0	
^99m^Tc-MAG3 (Renal dynamic)	<1	38.33	74.65	79.75	-
	1–<5	75.00	78.10	80.10	23 Kuwait [18], 76 Pakistan [19], 55 Saudi Arabia [21]
	5–<10	79.70	113.40	119.80	33 Kuwait [18], 95 Pakistan [19], 72 [20], 55 Saudi Arabia [21]
	10–<15	119.90	149.00	180.80	45 Kuwait [18], 152 Pakistan [19], 87 [20], 125 Saudi Arabia [21]
^99m^Tc-DMSA (Renal static)	<1	39.75	75.8	79.1	-
	1–<5	77.30	79.30	80.05	33 Kuwait [18], 164 Pakistan [19], 51 Saudi Arabia [21]
	5–<10	81.50	96.80	110.80	48 Kuwait [18], 177 Pakistan [19], 49 [20], 41 Saudi Arabia [21]
	10–<15	101.9	113.4	169.2	64 Kuwait [18], 198 Pakistan [19], 66 [20], 55 Saudi Arabia [21]

**Table 5:** CT DRLs based on weight bands used for attenuation correction and localization in hybrid PET/CT procedures compared with other countries.

PET/CT Protocol	Weight Band (kg)	CTDI_vol_ (mGy)	Other Countries	DLP (mGy.cm)	Other Countries
		25th, 50th (DRL), 75th Percentile		25th, 50th (DRL), 75th Percentile	
Tumor/Infection/Neuroendocrine, whole body scan	5–<15	1.52, 1.52, 1.52	0.57 Australia [17]	155.4, 173.3, 173.3	52 Australia [17]
	15–<30	1.52, 1.52, 1.63	0.81 Australia [17]	173.3, 191.2, 223.7	95 Australia [17]
	30–<50	2.0, 2.14, 2.91	1.08 Australia [17]	268.8, 297.8, 298.1	160 Australia [17]
	50–<80	2.86, 4.08, 5.59	1.95 Australia [17]	338.3, 488.7, 780.2	240.5 Australia [17]

**Table 6:** CT DRLs based on age band used for attenuation correction and localization in hybrid PET/CT procedures compared with other countries.

PET/CT Protocol	Age Band (Year)	CTDI_vol_ (mGy)	Other Countries	DLP (mGy.cm)	Other Countries
		25th, 50th (DRL), 75th Percentile		25th, 50th (DRL), 75th Percentile	
Tumor/Infection/Neuroendocrine, whole body scan	1–<5	1.52, 1.52, 1.52	0.81 Australia [17]	155.4, 173.3, 173.3	95 Australia [17]
	5–<10	1.54, 1.63, 1.95	1.08 Australia [17]	201.7, 223.7, 245.1	160 Australia [17]
	10–<15	1.99, 2.76, 3.84	1.95 Australia [17]	265.9, 313.2, 567.9	240.5 Australia [17]

## Data Availability

The original contributions presented in this study are included in the article. Further inquiries can be directed to the corresponding author.
